# Tracking of Magnetite Labeled Nanoparticles in the Rat Brain Using MRI

**DOI:** 10.1371/journal.pone.0092068

**Published:** 2014-03-14

**Authors:** Naira P. Martínez Vera, Reinhold Schmidt, Klaus Langer, Iavor Zlatev, Robert Wronski, Ewald Auer, Daniel Havas, Manfred Windisch, Hagen von Briesen, Sylvia Wagner, Julia Stab, Motti Deutsch, Claus Pietrzik, Franz Fazekas, Stefan Ropele

**Affiliations:** 1 Department of Neurology, Medical University of Graz, Graz, Austria; 2 Institute of Pharmaceutical Technology and Biopharmacy, University of Muenster, Muenster, Germany; 3 JSW-Live Sciences GmbH, Grambach, Austria; 4 Department of Cell Biology & Applied Virology, Fraunhofer Institute for Biomedical Engineering, St. Ingbert, Germany; 5 Physics Department, Schottenstein Center for the Research and Technology of the Cellome, Bar Ilan University, Ramat Gan, Israel; 6 Institute of Pathobiochemistry, University Medical Center of the Johannes Gutenberg-University Mainz, Mainz, Germany; CINVESTAV-IPN, Mexico

## Abstract

This study was performed to explore the feasibility of tracing nanoparticles for drug transport in the healthy rat brain with a clinical MRI scanner. Phantom studies were performed to assess the R_1_ ( =  1/T_1_) relaxivity of different magnetically labeled nanoparticle (MLNP) formulations that were based on biodegradable human serum albumin and that were labeled with magnetite of different size. In vivo MRI measurements in 26 rats were done at 3T to study the effect and dynamics of MLNP uptake in the rat brain and body. In the brain, MLNPs induced T_1_ changes were quantitatively assessed by T_1_ relaxation time mapping in vivo and compared to post-mortem results from fluorescence imaging. Following intravenous injection of MLNPs, a visible MLNP uptake was seen in the liver and spleen while no visual effect was seen in the brain. However a histogram analysis of T_1_ changes in the brain demonstrated global and diffuse presence of MLNPs. The magnitude of these T_1_ changes scaled with post-mortem fluorescence intensity. This study demonstrates the feasibility of tracking even small amounts of magnetite labeled NPs with a sensitive histogram technique in the brain of a living rodent.

## Introduction

The use of nanoparticles (NPs) is a promising approach to deliver therapeutics into the brain of subjects with central nervous system (CNS) disorders [1]–[3]. A first and important step in this endeavor lies in the investigation of the kinetic and biological behavior of nanostructures in vivo. It has been shown that NP uptake including blood brain barrier (BBB) transition and their processing strongly depends on their size, shape, surface charge and surface coating [4]–[7]. While a lot of recent research has focused on the bio-distribution of numerous classes of nanoparticle, further investigation is needed to know about the transition of intravenously administered NPs across the BBB and how the NPs are uptaken by the mononuclear phagocyte system [6]–[11].

Magnetic resonance imaging (MRI) carries a high potential to localize NPs within the body in vivo because of its high spatial resolution and non-invasive nature. However, because protein based NPs do not induce intrinsic MRI contrast, they need to be labeled with paramagnetic or superparamagnetic nanomaterials (magnetically labeled nanoparticles, MLNPs) [6], [12] or with nanomaterial that exhibits a chemical shift [13]–[17] in order to study NP organ delivery by MRI. Contrast enhancing magnetic nanoparticles provide a high magnetic susceptibility which can produce strong microscopic field gradients. Consequently, dipole-dipole interactions accelerate T_1_ relaxation (positive contrast) while dephasing effects in the field gradients accelerate T2* relaxation, i.e. the effective loss of the transversal magnetization of the free induction decay (negative contrast). Magnetite (Fe_3_O_4_) is a ferrimagnetic compound. However, spherical magnetite nanoparticles under some conditions such as a small diameter (< 20 nm) exhibits superparamagnetic behavior [18]. Superparamagnetic particles have no net magnetization if no external field is applied but its magnetic saturation is comparable with ferromagnetic particles [6], [12], [18], [19]. The quantitative assessment of negative contrast effects using small animals is more difficult on clinical scanners because of the faster signal decay. In practice, the sensitivity for probing smallest amounts of MLNPs is limited by the relaxivity of the MLNPs and by the sensitivity of the MRI system which is a complex function of several factors including field strength, coil sensitivity and pulse sequence. Current developments therefore focus on improved MRI acquisition and post-processing methods with the aim of a better image contrast and shorter acquisition time [12], [20]–[24].

In this explorative study, we tested different MLNP formulations constructed of human serum albumin (HSA) and magnetite which can incorporate drugs. This protein-based class of NPs is a very promising future tool for medical substance delivery and in vivo tracking, since the NPs are biodegradable and non-antigenic [25]–[28]. After identifying the most promising candidate, i.e. the one with strongest relaxivity effect for in vivo imaging, MLNPs were administered intravenously in rats to study their kinetics in the body and the brain. As no specific accumulation was expected and no modifications were made to facilitate BBB transition, we developed a robust and sensitive histogram technique to assess even smallest MLNP induced T_1_ changes in the brain. Finally, these changes were substantiated and validated with histogram analyses of fluorescence obtained from post-mortem tissue.

## Materials and Methods

All MR experiments were performed on a conventional clinical 3.0 T scanner (Tim Trio, Siemens Healthcare, Germany) using a 2-element surface coil for signal reception.

An Axio Imager Z1 microscope (Carl Zeiss AG, Germany) served to image MLNP fluorescence using a 10x Plan Apochromat lens (NA 0.45) and an Axio mRM high sensitive camera with 1x opto-coupler. The camera was set to the linear default in AxioVision (v4.8) software control. The filter sets used were Fs38HE and Fs43 (https://www.micro-shop.zeiss.com/us/us_en/spektral.php).

### Ethics statements

Animal studies conformed to the Austrian guidelines for the care and use of laboratory animals and were approved by the Styrian Government (FA10A-78Jo-98-2012).

### Synthesis of magnetite labeled HSA nanoparticles

Human serum albumin (20 mg) was dissolved in 2 mL purified water and the pH was adjusted to 8.0. The solution was filtered through a 0.22 μm cellulose acetate membrane filter. An aliquot (1 mL) of this solution was mixed with 33.3 μL (1 mg) magnetite dispersion with an average particle size of 8 nm (PlasmaChem, Germany) and 10 μL 1 M NaCl solution. The mixture was incubated for 1 h at 20°C under constant stirring (600 rpm). To initiate nanoparticle formation, 4 mL ethanol were added using a tubing pump. Particles were stabilized by crosslinking using 5.8 μL glutaraldehyde solution (8%). The crosslinking was performed for at least 12 h under constant stirring at room temperature. A similar procedure was repeated with the different particle sizes of magnetite (diameter of 20 nm and 8 nm) and magnetite loading (47.62 μg/mg MLNP and 90.9 μg/mg MLNP). After preparation the particles were purified by three centrifugation steps (10,000 g, 5 min) and redispersion of the pellets to the original volume in purified water.

### Preparation of PEGylated nanoparticles

For the in vivo studies, some of the MLNPs were additionally modified with polyethylene glycol (PEG). Different studies demonstrated that coating their surface with different hydrophilic surfactants such as PEG polymer increases MLNP blood circulation time thus avoiding their fast clearance by the mononuclear phagocyte system [29], [30].

For PEGylation magnetite labeled HSA nanoparticles were prepared as described above and modified as follows: 1 ml of HSA NP (20 mg/ml) was incubated with 300 μl methoxy PEG succinimidyl active ester (mPEG-SSA, Rapp Polymere, Germany) solution (85 mg/ml in phosphate buffer pH 8.0) for 1 h at 20°C under constant shaking (600 rpm, Eppendorf thermomixer). The nanoparticles were purified by centrifugation and redispersion as described above.

### Nanoparticle characterization

Nanoparticles were analyzed with regard to particle diameter and polydispersity by photon correlation spectroscopy (PCS) using a Malvern Zetasizer Nano ZS (Malvern Instruments Ltd., Malvern, UK). Prior to measurement the samples were diluted with purified water.

The concentration of the purified nanoparticles was determined by microgravimetry. An aliquot (20.0 μl) of the respective nanoparticle sample was put in a micro weighing dish (VWR, Germany). After a drying time of 2 h at 80°C the dish was weighted with a microbalance (Sartorius, Germany). The content of the nanoparticles was calculated from the difference of the empty and nanoparticle filled dish.

The amount of incorporated magnetite was investigated by total reflection X-ray fluorescence (TXRF) in the Group of Prof. Karst (University of Muenster, Institute of Inorganic and Analytical Chemistry).

Scanning electron microscopy (SEM) images of the magnetite labeled nanoparticles were performed by Elektronen-Optik-Service GmbH, Germany.

### Phantom studies

To assess the T_1_ relaxivity of different NP formulations, T_1_ mapping was performed in six small samples containing different concentrations (0.039%, 0.078%, 0.156%, 0.313%, 0.625%, and 1.25%) of HSA based MLNPs in 2 mL of solution. T_1_ was determined with an inversion recovery sequence (repetition time (TR)  =  5000 ms, echo time (TE)  =  9.2 ms) with multiple inversion times (TI  =  50, 100, 200, 400, 800, 1600, 3200 ms). T_2_* was not assessed due to the limited readout bandwidth.

### In vivo studies

**Figure 1:**
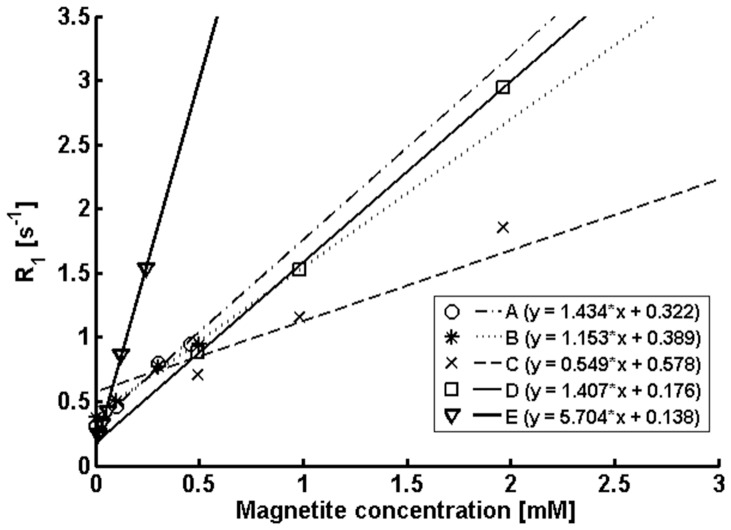
Effective relaxation rate constant R_1_ ( =  1/T_1_) as a function of magnetite concentration of five different MLNP formulations. The letters correspond to the formulations in Table 1.

To study the accumulation of NPs in the brain and in other organs, in vivo experiments were performed in 12-30 weeks old female Wistar wild rats (n  =  26). MLNPs with a magnetite diameter of 8 nm and magnetite concentration of 90.9 μg/mg MLNP showed the strongest R_1_ relaxivity effect in the phantom measurements (Figure 1) and therefore were used for these experiments. Before MRI, rats were pre-sedated with isoflurane inhalation anesthesia. Sedation was done with a mixture of ketamine and xylazine (2.3:1) where 1.66 μL per gram body weight were injected intraperitoneally.

Two rats served to explore the accumulation of MLNPs in the body following intravenous injection of 400 μL (800 μg) MLNPs through the femoral vein. Imaging was done with a phase sensitive inversion recovery sequence (TI  = 100 ms; TE  = 9.2 ms; TR = 5000 ms; slice thickness (THK)  = 1.44 mm; field of View (FOV)  = 5 mm; matrix  = 180×256×20) which provides improved T_1_ contrast. The sequence was performed before NP administration and 9, 18, 27, 36 minutes after the injection. No physiological triggering was employed, however a saturation slab was applied on the lungs to reduce breathing artifacts. In another rat, 400 μL MLNPs were administered by intraventricular injection to achieve a higher MLNP concentration in the brain.

In 23 rats we focused on the uptake of MLNPs in the brain. In these cases, T_1_ in the brain was estimated with a 3D mode acquisition and driven equilibrium single pulse observation of T_1_ (DESPOT, flip angle (FA)  = 6° and 30°, TR = 30 ms; THK  = 1 mm; FOV  =  40 mm; matrix  =  192×192×32) [31]. To increase the signal to noise ratio (SNR) of the T_1_ maps, the sequence was performed with two echoes (TE_1_  =  4.82 ms, TE_2_  =  9.96 ms) and with 3 repetitions (total scan time for T_1_ determination  =  26 min). T_1_ maps were calculated for both echoes separately before averaging. To reduce flip angle errors due to slice profile effects, a non selective excitation pulse was used. Additionally, a saturation slab was placed caudally to suppress residual signal from the rat body and to prevent backfolding. As this study focused on the longitudinal changes of T_1_ and therefore was based on serial T_1_ mapping, no attempts were made to correct for B_1_ induced T_1_ errors. Following baseline T_1_ mapping, MLNPs were injected intravenously into the vena femoralis without removing the rat from the coil through a catheter that had been prepared before MRI. A 4.8 mg of MLNP vial with 1 mL physiological buffer was used to suspend MLNPs, which were administered at 3.2 μL per gram body weight. Saline solution at equal volume served as control in 7 out of the 23 rats. After injection, T_1_ mapping was subsequently repeated two times. In the group of rats with MLNP administration, 11 rats received PEG coated MLNPs. A rectal probe continuously monitored the body temperature of the rats during MRI (LUXTRON 790 Fluoroptic Thermometer, LumaSense Technologies, Santa Clara, CA, USA).

### Assessment of T_1_ changes

**Figure 2:**
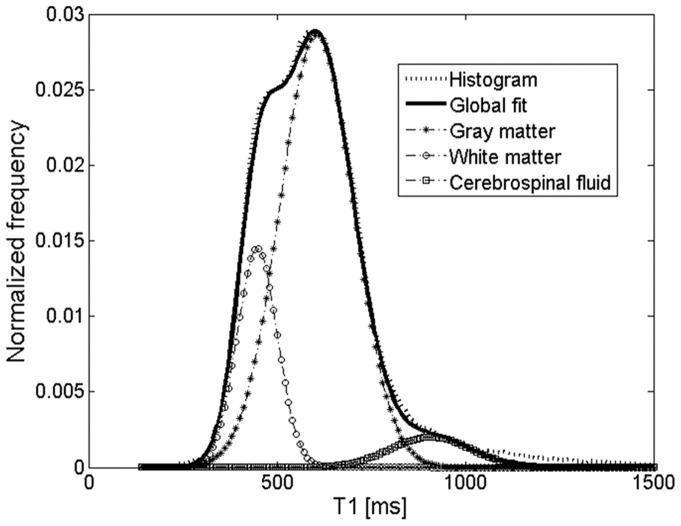
T_1_ histogram (dotted line) of a rat brain. Triple Gaussian fitting allowed for separation of white matter (circles), gray matter (diamonds) and cerebrospinal fluid (squares).

Following generation of T_1_ maps, brain tissue was manually segmented (FSLview tool: http://fsl.fmrib.ox.ac.uk/fsl/fslview/) and globally analyzed with a histogram technique using a home written script in Matlab (The MathWorks, Natick, MA, USA). The T_1_ distribution in the entire brain was modeled by fitting a triple Gaussian function with a least-square technique. The three Gaussians represented gray matter (GM), white matter (WM), and cerebrospinal liquid (CSF) (Figure 2). The peak positions were used as the global T_1_ value for these structures which facilitated robust analysis of MLNP induced relaxivity changes. The rationale behind this approach was an expected global and diffuse distribution of MLNPs in the brain (diffusion model) rather than an accumulation in specific structures.

### Fluorescence imaging

Immediately after MRI the rats were sacrificed. Rats were transcardially flushed with 0.9% saline to remove eventual NPs within vessels together with red blood cells, both of which would bias signal measurements. The brains were then extracted and fixed in a 4% neutral buffered paraformaldehyde (PFA) solution for two hours to stabilize proteins and the albumin of the NPs. After cryo-protection in 15% sucrose until sunk, hemibrains were frozen in dry ice cooled liquid isopentane and systematically cut sagittally at 16μm thickness on a Leica CM3050 S cryotome. Short PFA exposure guarantees a low green autofluorescent background in the slices but sufficiently fixes the tissue. Air dried and embedded in Mowiol, mosaic images from the corpus callosum (CC) and the hippocampus (HC) were recorded using equal exposure times for each channel and highly constant LED excitation (“Colibri” on a Zeiss Z1 AxioImager with sensitive mRM B&W camera and 10x Plan Apochromate lens). Histogram measurements were performed with ImageProPlus software (v6.2) after manual delineation of the region of interest (ROI). Histogram measurements of three different medio-sagittal brain levels (approximately 0.2 mm, 1 mm and 2 mm lateral) were averaged per region to one individual mean per animal. Mean pixel intensities of the CC and HC were assessed for the red and green channel. To evaluate MLNP uptake in the rest of the body, liver and spleen were sampled, cut and investigated for direct MLNP fluorescence of microscopically visible accumulations.

An additional experiment in a single rat was performed to detect and validate the MLNP related fluorescence [32], [33] by comparing fluorescence imaging and Prussian blue labeling to Fe^3+^ (Hematognost Fe, Merck). Dispersed and diffused MLNP are below the microscopic resolution. To achieve a significant accumulation of MLNP deposits and to prove their direct detectability with the used setup, MLNPs were administered into the lateral ventricle and imaged in consecutive sections, one for direct fluorescence, the second stained for Prussian Blue in brightfield.

### Statistical analysis

A linear regression analysis (Pearson’s r) was performed to study the relationship between MLNP induced T_1_ changes and relative levels of fluorescence. A paired t-test was used for comparison of ex vivo signal as well as to determine significant T_1_ changes following MLNP injection, i.e. to verify whether minimal uptake of MLNPs can be probed by quantitative MRI. A p-value of <0.05 was considered as statistically significant.

## Results

### NP preparation and characterization

**Figure 3:**
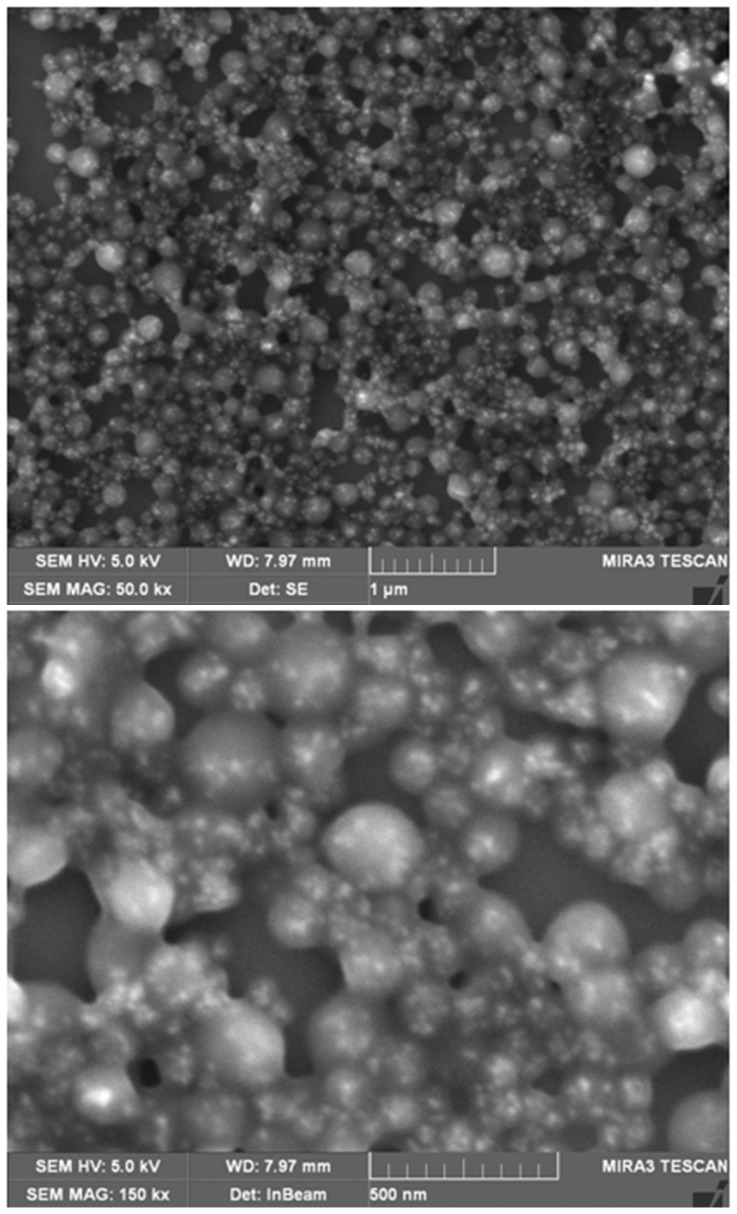
SEM images (Tescan MIRA XMU with YAG BSE-detector and SE-detector) of HSA nanoparticles labeled with magnetite (8 nm). The light spots show the incorporated magnetite. Upper image: 20,000-fold magnification with 5.78 μm field of view, lower image: 60,000-fold magnification with 1.93 μm field of view.

In this study we prepared magnetite labeled HSA nanoparticles with a particle diameter between 200 and 550 nm. The samples showed monodisperse (PDI <0.1) or slightly polydisperse (PDI <0.2) size distribution (Table 1). The amount of incorporated magnetite was calculated by TXRF and indicated almost quantitative magnetite entrapment during the preparation of the nanoparticles (data not shown). SEM images of MLNPs confirmed the results of the PCS and TXRF experiments with regard to particle size and incorporation of magnetite (Figure 3). Furthermore, in earlier cell culture experiments we could show that nanoparticles based on HSA were non-toxic in concentrations up to 1000 μg/ml [26], [28], [34].

**Table 1:** Relaxivity of the different magnetically labeled nanoparticle formulations.

Letter series	Diameter of MLNP [nm]	Diameter of magnetite [nm]	Polydispersity index (PDI)	Magnetite concentration [μg/mg MLNP]	r_1_ ± standard deviation [mM^−1^s^−1^]
A	462.8	20	0.17	47.62	1.43±0.11
B	551.7	20	0.03	47.62	1.15±0.08
C	201.6	20	0.04	90.90	0.54±0.06
D	318.1	20	0.12	90.90	1.40±0.33
E	200.8	8	0.09	90.90	5.70±0.23

### Phantom measurements

Results from the phantom measurements with different MLNP concentrations are summarized in Figure 1. In line with theoretical considerations, the longitudinal relaxation rate constant R_1_ ( =  1/T_1_) was found to correlate linearly with magnetite concentration. The size of the MLNPs showed some effect on the relaxivity; however, the slope of the regression line was mainly determined by the size of the magnetite. The strongest T_1_ effect was found for the MLNPs with a mean diameter of approx. 200 nm and an 8 nm magnetite loading of 90.90 μg per mg of MLNP (Table 1).

### In vivo studies

**Figure 4:**
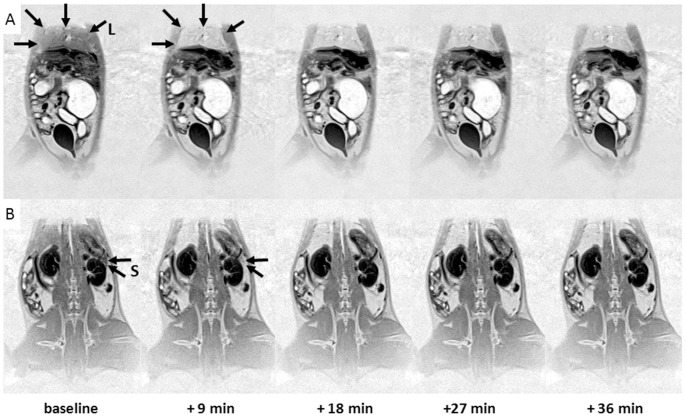
Uptake of MLNPs in the rat body. Two coronal MRI slices through the rat body are shown. Following MLNP injection, a significant and long lasting signal increase (arrows) was mostly observed in the liver L (row **A**) and in the spleen S (row **B**).

**Figure 5:**
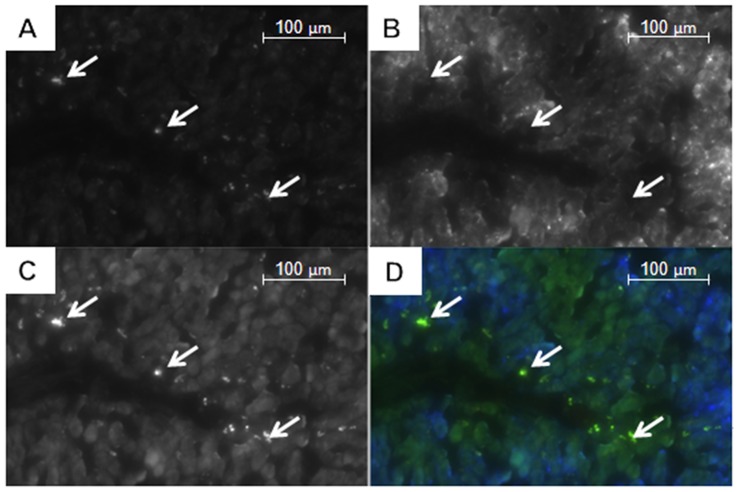
Uptake of MLNPs in the liver. Fluorescence images from a piece of the left liver lobe (bile duct) obtained from the red (**A**), blue (**B**), green (**C**), and the sum of these three channels (**D**). Only MLNP agglomerations (arrows) are best seen in the green and red channel with the latter having the lowest background fluorescence.

In the body of the rat, the phase sensitive inversion recovery sequence provided sufficient resolution and contrast to assess MLNP induced signal intensity changes. Most pronounced changes presented as an increase in signal intensity were observed in the liver and in the spleen. These changes were apparent at the first imaging time point 9 minutes after MLNP injection and thereafter showed almost no further changes over the observational period of 36 minutes (Figure 4). In parallel to MRI, these regions also presented marked changes in the fluorescence signal (Figure 5).

**Figure 6:**
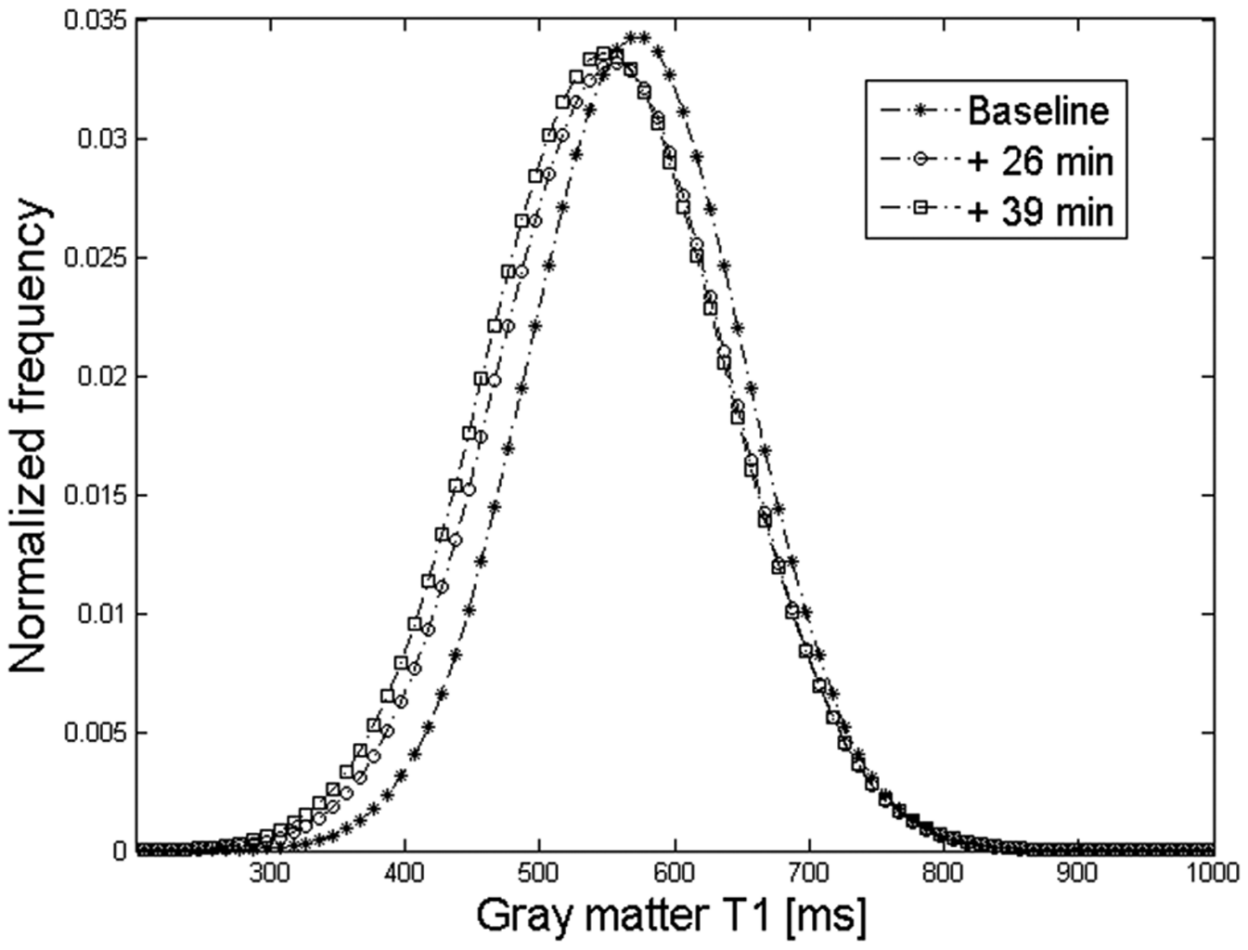
Histogram analysis revealed a significant MLNP induced shift of the gray matter peak towards shorter T_1_ values after 26 and 39 minutes of the injection.

**Figure 7:**
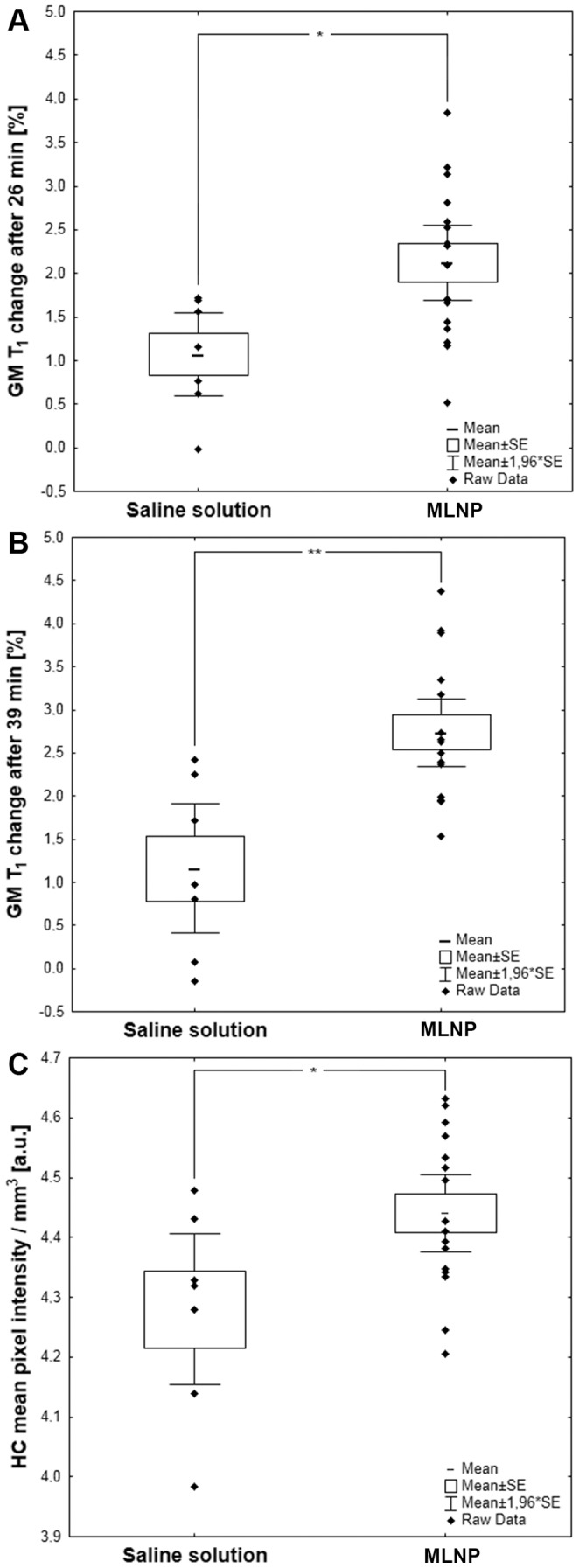
MLNP uptake corresponded to significant T_1_ shortening and an increase in fluorescence signal intensity. GM T_1_ values in rats treated with MLNPs were significantly decreased in comparison with control group after 26 minutes (**A**) and after 39 minutes (**B**). (**C**) The fluorescence signal in the hippocampus (HC) was significantly increased in the red channel after MLNP administration when compared to the control group (saline solution instead of MLNPs). * p<0.05; ** p<0.01.

**Figure 8:**
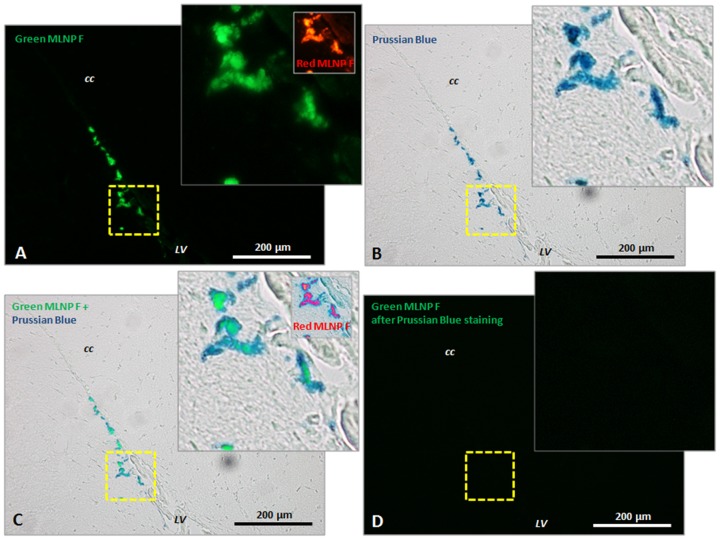
Prussian Blue staining of fluorescencing MLNP accumulations close to the intracerebroventricular injection site. (**A**) The magnetite in MLNPs shows emission in the green and red (insert) spectrum with blue and green excitation. It was imaged before doing the Prussian Blue staining from an untreated cryo-slice from PFA fixed brain. (**B**) shows the Prussian Blue staining, whereas blue color derives from the hexacyanoferrat complex. (**C**) is a maximum difference projection of (**A**) and (**B**), showing the overlap of MLNP fluorescence and Prussian Blue staining. (**D**) The fluorescence totally vanishes after Prussian Blue staining, indicating that binding the iron in the complex alters the excitability of the magnetite molecules. Abbreviations: F  =  fluorescence; LV  =  lateral ventricle; CC  =  corpus callosum. Magnification  =  20x.

**Figure 9:**
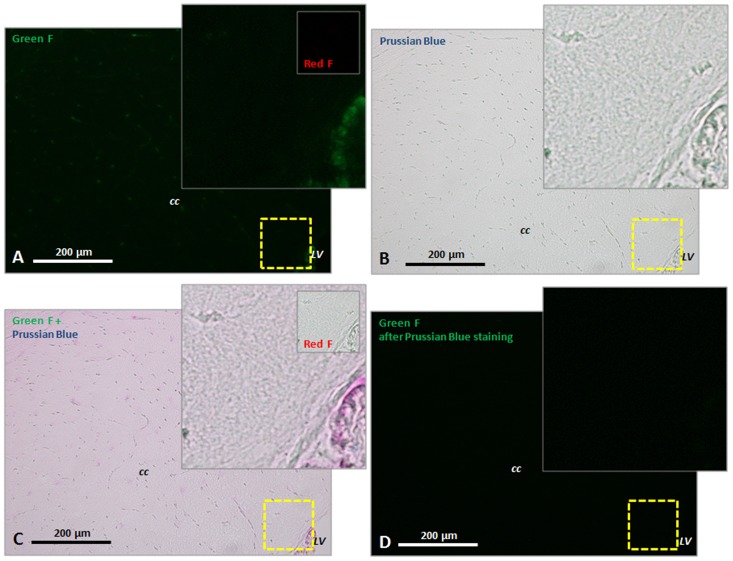
Prussian Blue staining close to the injection site of the rat shown in Figure 8 in a vehicle control rat. (**A**) Slice fluorescence levels are generally low in the cryo slices and in most cases restricted to vasculature as shown here for some ependymal cells of the chorioid plexus. (**B**) shows the Prussian Blue staining, whereas in healthy rats iron accumulations (from micro bleedings) in the brain are typically absent (**C**) is a maximum difference projection of (**A**) and (**B**). (**D**) The slice fluorescence vanishes during Prussian Blue staining and brightfield imaging. Abbreviations: F  =  fluorescence; LV  =  lateral ventricle; CC  =  corpus callosum. Magnification  =  20x.

In the brain, T_1_ histogram analyses revealed a significant MLNP induced shift of the GM and WM peaks towards shorter T_1_ values (Figure 6, and Figure 7A and B). In parallel, the mean fluorescence signal related to MLNPs increased significantly in the investigated rat brain sections of the CC and HC in the green and red channels (Figure 7C). Only very high concentrations of MLNPs can be microscopically seen on fluorescence imaging such as those achieved after intraventricular injection. Figure 8 shows the accumulations of agglomerated MLNPs following direct intraventricular injection in a single rat close to the intracerebroventricular injection site. The localization in the corresponding Prussian Blue iron stain indicates that the fluorescence of the MLNPs is induced by the magnetite. A direct co-imaging is not possible, since the excitability of the magnetite disappears when the iron is in the Prussian blue complex. In contrast, control rats showed lower fluorescence levels (Figure 9A) and iron staining of corresponding slices did not reveal any accumulation of iron (Figure 9B).

**Figure 10:**
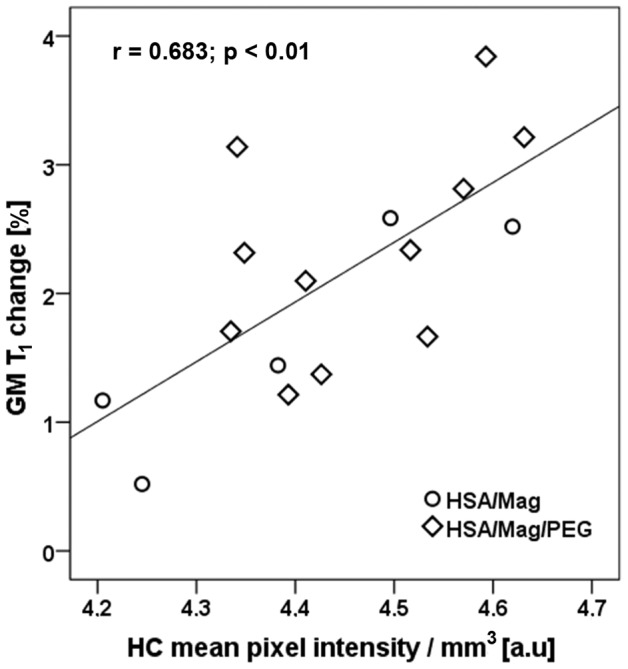
T_1_ changes in gray matter (GM) of the 16 MLNP treated rats versus fluorescence signal in the hippocampus (HC) of the red channel.

Table 2 provides the results of the linear regression analysis between T_1_ changes and results from fluorescence imaging. In all regions assessed, MLNP induced T_1_ shortening scaled with the relative fluorescence signal with the red channel showing the highest sensitivity_._ Exemplarily, Figure 10 shows the relationship between the T_1_ changes in GM and the fluorescence signal in the HC of the red channel. Overall, the correlations were better for the first T_1_ measurement following MLNP injection (Table 2) than at the end of the experiment. Temperature measurement showed that the mean temperature in rat body decreased by 2.7°C between the start and end of the brain MRI examination.

**Table 2:** Relationship between MLNP induced T_1_ changes in white matter (WM) and gray matter (GM) and fluorescence signal in the red channel.

	T_1_-WM_1_	T_1_-WM_2_	T_1_-GM_1_	T_1_-GM_2_
Fluorescence in				
corpus callosum (CC)	0.675**	0.490*	0.640**	0.625**
hippocampus (HC)	0.612*	0.495*	0.683**	0.610*
CC + HC	0.649**	0.495*	0.664**	0.622*

The Pearson correlation coefficient is shown (* p<0.05; ** p<0.01). The subscripts 1 and 2 correspond to the post injection delay of 26 and 39 min, respectively.

## Discussion

This study demonstrates that magnetite labeling enables in vivo tracing of the delivery of NPs to different organs in a rat model MRI. Results from phantom studies suggest that the relaxivity of the MLNPs is high enough to allow detection of even low concentrations in vivo. In agreement with relaxation theory, it could be shown that R_1_ linearly increases with MLNP concentration but the relaxivity is mostly determined by the size of the magnetite. A smaller size of paramagnetic particles also increases the R_1_ relaxivity while R_2_* relaxivity increases with particle size [35]. Given the limited SNR and readout bandwidth provided by a clinical scanner, this was also the reason why we focused on T_1_ effects rather than on T_2_* effects in the current investigations. Quantitative assessment of T_2_* needs a gradient echo sequence with at least two echoes, but the minimum echo times resulting from a limited readout gradient at a small field of view may not be optimal for assessing regions with a high MLNP concentration. Moreover, we did not look for focal and macroscopically visible effects of MLNP accumulation but rather for global effects.

While the accumulation of MLNPs was clearly visible in some organs, visual inspection failed to discover MLNP uptake in the brain. This was not unexpected because the BBB usually blocks compounds of the size administered in current study. Some studies [36], [37] showed that after injection of PEGylated HSA nanoparticles only a few were visible in the endothelial cells of the BBB, suggesting that these NPs are unselectively retained within the endothelial cells, possibly within the lysosomal compartment. However, the histogram technique proposed in our work was capable to demonstrate a subtle but global T_1_ change in the gray and white matter brain tissue compartments. Using a triple Gaussian model, the three major tissue components could be well delineated (Figure 2). There was a dominant GM peak representing contributions from the cortex and basal ganglia structures, followed by a small peak representing WM, and an additional peak reflecting contributions from CSF. The detection limit for the amount of MLNPs is difficult to predict because it depends on the size of the compartment or region, i.e. number of voxel included, and the overall signal-to noise ratio. However, a very rough estimation can be made from the experimentally derived relaxivity and the uncertainty of the T_1_ estimation, i.e. standard deviation of the Gaussian histogram peak, provided that the nanoparticles are homogenously distributed in a tissue compartment. In our setting with a standard error of approximately 6%, a significant T_1_ shift in the histogram can be observed if the accumulation of nanoparticles exceeds 0.015 mg/L magnetite. The kinetics of the HSA based MLNPs is not fully clear but it seems that accumulation in the liver, spleen and brain occurs at high rate and that the time constant of the clearing rather long. In this context, it is important to note that MRI just probes the presence of magnetite in brain tissue. Thus, our experiments cannot exclude the possibility that MLNPs might have been degraded despite the occurrence of MR signal changes. Given the rather small decrease of T_1_, it can be concluded that only a very low percentage had entered the brain. However, it is not possible from our data to separate between nanoparticles that really crossed BBB and nanoparticles that get stuck in the vascular endothelia. Although the observed T_1_ changes were small, they correlated linearly with the signal intensity derived by fluorescence imaging which attests the high sensitivity of our imaging and analysis approach. Whether the fluorescence signal from MLNPs stems from the iron or also from the serum albumin is not fully clear but the validation with control rats and Prussian blue staining where the binding of the iron in the complex alters the excitability of the magnetite molecules, suggest that the magnetite is responsible for the high fluorescence signal. Significant correlations were found for all regions, but the best correlation was found between the signal in the HC and the T_1_ change in GM. This may be explained by the fact that GM exhibits a higher blood volume thus allowing for more pronounced MLNP effects. Additionally, the green fluorescence background signal was generally higher in all compartments at equal exposure time, which is due to natural emission [38], but also PFA fixation that enhances green fluorescence. This may explain why a higher correlation was found for the red channel.

Interestingly, the correlation coefficients between MRI and fluorescence signal were generally lower for the second T_1_ measurement, i.e. the one that was performed before sacrificing the animals and therefore should be more closely related to the ex vivo data. A possible explanation for this observation is that we did not consider the effect of cooling of the brains. Due to anesthesia, body temperature of the rats dropped approximately 10% over the span of the MRI experiments. As T_1_ depends on brain temperature [39], [40], the anesthesia induced a temperature drop that has an effect in the same direction as the uptake of MLNPs and therefore may have affected the correlation analysis. We were not able to correct for the temperature effect, because we found no significant relationship between brain T_1_ and temperature in the rectum. Further experiments should therefore consider this effect either by an appropriate temperature correction of T_1_, or by using a heating pad to keep the rat body at constant temperature.

## Conclusions

In conclusion, we have demonstrated that NPs can be magnetically labeled such that they are traceable by MR T_1_ relaxometry. Despite the use of a clinical MRI scanner with limited sensitivity, we were able to detect diffuse and global accumulation of MLNPs by the implementation of a new histogram based technique. To achieve higher rates of brain MLNP uptake, further research is needed to determine ways of MLNP modification which promote MLNP transport over the BBB. Respective investigations are under way as for example our research group has started experiments on MLNP modification with apolipoprotein E [36], [37], [41].
